# Effects of Jian Pi Qing Chang Hua Shi decoction on mucosal injuries in a 2,4,6-trinitrobenzene sulphonic acid-induced inflammatory bowel disease rat model

**DOI:** 10.1080/13880209.2021.1928240

**Published:** 2021-06-10

**Authors:** Huicun Zhang, Na Ta, Hong Shen, Hongbing Wang

**Affiliations:** a Beijing Hospital of Traditional Chinese Medicine, Capital Medical University, Beijing, China; b Beijing Institute of Chinese Medicine, Beijing, China; c Center Hospital of Beijing Daxing District Caiyu Town, Beijing, China; d Jiangsu Province Hospital of Traditional Chinese Medicine, Nanjing, China; e Beijing Hospital of Traditional Chinese Medicine Yanqing Hospital, Beijing, China

**Keywords:** Superoxide dismutase, malondialdehyde, myeloperoxidase, interleukin-1β, tumour necrosis factor-α, immunoglobulin A, nuclear factor-κB

## Abstract

**Context:**

Jian Pi Qing Chang Hua Shi decoction (JPQCHSD) has been considered as an effective remedy for the treatment of inflammatory bowel disease (IBD) in Chinese traditional medicine.

**Objective:**

We evaluated the efficacy of JPQCHSD on 2-4-6-trinitrobenzene sulphonic acid (TNBS)-induced IBD rats and the responsible mechanisms.

**Materials and methods:**

Except the rats of the control group (50% ethanol), Sprague–Dawley rats (180 ± 20 g) induced by TNBS (150 mg/kg in 50% ethanol), received water extract of JPQCHSD daily at 0, 9.5, 19, or 38 g/kg for 12 days. The rats were sacrificed, and their colons were removed to evaluate the disease activity index. Malondialdehyde (MDA), superoxide dismutase (SOD), myeloperoxidase (MPO), immunoglobulin A (IgA), tumour necrosis factor (TNF)-α, interleukin (IL)-1β, IL-6, and nuclear factor-κB were evaluated.

**Results:**

JPQCHSD extract significantly reduced the disease activity index of TNBS-induced colitis with a median effective dose (ED_50_) of 26.93 g/kg. MPO and MDA were significantly reduced in the 19 and 38 g/kg groups (ED_50_ values 37.38 and 53.2 g/kg, respectively). The ED_50_ values for the increased SOD and IgA were 48.98 and 56.3 g/kg. ED_50_ values for inhibition of TNF-α, IL-1β, and IL-6 were 32.66, 75.72, and 162.06 g/kg, respectively.

**Discussion:**

JPQCHSD promoted mucosal healing in IBD rats via its anti-inflammation, immune regulation, and antioxidation properties.

**Conclusions:**

JPQCHSD has healing function on IBD. Further clinical trials are needed to demonstrate its efficacy and tolerance to IBD.

## Introduction

Inflammatory bowel disease (IBD), which includes Crohn’s disease and ulcerative colitis, is a chronic inflammatory disease characterized by chronic and recurrent intestinal inflammation. IBD is caused by many factors, such as environmental stress, and is widely accepted to have a genetic component with individual susceptibility enhanced by an ongoing mucosal immune response (Seidelin 2015). The aetiology and molecular mechanism of the pathogenesis of IBD remains unclear. Therefore, conservative treatment of IBD is controversial and effective measures to prevent recurrence are lacking.

IBD has been recognized as a modern refractory disease associated with cellular immunity, oxidative stress, and autoimmunity. Immune and oxidative stress dysfunctions play important roles in the pathogenesis of IBD. Oxygen-free radicals are released in inflammatory areas via the action of inflammatory stimulants. When oxygen-free radical levels exceed the scavenging capacity of the body, they damage surrounding tissues. This promotes a vicious cycle between inflammation and tissue damage (Roessner et al. 2008; Feuerstein et al. 2019).

Nuclear factor (NF)-κB is a nuclear protein that binds to the promoter sites of many cellular genes *in vivo*. It plays an important role in immune, inflammatory, and apoptotic regulation. The activation of NF-κB can cause various pathophysiological reactions. Overactivation of NF-κB in IBD leads to excessive and persistent production of cytokines. Continuous expression of NF-κB enhances the inflammatory response; thus, NF-κB participates in intestinal inflammatory and immune responses in IBD (Wang et al. 2019). The oxidative stress pathway is important in the regulation of NF-κB (Menghini et al. 2016).

Presently, drugs commonly used for treating IBD include immunosuppressants and corticosteroids. However, most of these drugs only relieve symptoms temporarily and cause slight to serious side effects, limiting their clinical application (Thurgate et al. 2019; Barnes et al. 2020). Therefore, the effectiveness and safety of traditional Chinese medicine (TCM) formulations have attracted increased attention for treating IBD (Zhang et al. 2013).

According to TCM theory, the pathogenic characteristics of IBD are spleen and stomach damage induced by multiple pathogenic factors, which lead to dysfunction in the import and export of necessary molecules by these organs (Gong et al. 1995; Liu and Cai 2007; Duan et al. 2015). Damp and hot conditions, the Qi, and blood negatively interact in the large intestine, leading to the stagnation of Qi and blood, resulting in destruction of the large intestine. Under these conditions, IBD occurs. Sijunzi decoction (SJZDS) is a traditional Chinese herbal recipe for invigorating the spleen, stomach, and intestine that restores the imbalance of Qi and has been used in TCM clinics for a long time (Zhang et al. 1999; Wu and Xuan 2007; Tian et al. 2012, 2015; Lu et al. 2017, 2018).

SJZD repairs damage to the intestinal mucosa caused by 2,4,6-trinitrobenzene sulphonic acid (TNBS) in rats by regulating the intestinal barrier (Lu et al. 2017, 2018). To adjust the Qi, promote blood circulation, and eliminate dampness, we added other herbal medicines to SJZD. We named this mixture as the Jian Pi Qing Chang Hua Shi decoction (JPQCD). The herbs contained in JPQCHSD and their own traditional use of each component of JPQCHSD are listed in Tables 1 and 2. The Latin name of the herbs in in this paper are derived from Chinese Pharmacopoeia (2015). Lancemaside A is one of the components of *Codonopsis pilosula* (Franch.) Nannf (Campanulaceae). Lancemaside A improve TNBS-induced colitis by suppressing increased myeloperoxidase (MPO) activity, expression of inflammatory cytokines, and activation of NF-κB in mice (Joh et al. 2010). *Salvia miltiorrhiza* Bge (Labiatae) is a traditional Chinese medicine that has been long used for the treatment of cardiac cerebral diseases including cardiovascular diseases, neurodegenerative diseases, diabetes, nephropathy, liver, lung, skin, etc. Its clinical effects were by activating nuclear factor erythroid 2-related factor 2 (Li et al. 2018). *Atractylodes macrocephala* Koidz (Compositae) inhibits the inflammatory response of RAW 264.7 macrophages stimulated by lipopolysaccharide (LPS) by reducing key molecules in the mitogen-activated protein kinase (MAPK) and NF-κB pathways (Zhou et al. 2019). Coptisine inhibits NF-κB, MAPK, hosphoinositide 3-kinase/Akt activation and reduces inflammation induced by macrophages stimulated by LPS. It also is used to prevent and treat carrageenan-elicited rat paw oedoema and reduce the release of TNF-α and NO in rat inflamed tissue (Wu et al. 2016). *Smilax glabra* Roxb (Liliaceae) inhibits upper genital tract inflammation by blocking activation of the NF-κB pathway (Zou et al. 2017). *Sanguisorba officinalis* Linn (Rosaceae) exerts anti-inflammatory effects, making it useful for treating inflammatory skin diseases. It blocks NF-κB, signal transducer and activator of transcription 1 and extracellular signal-regulated kinase activation and inhibits tumour necrosis factor (TNF)-α/interferon-γ-stimulated release of chemokines and pro-inflammatory molecules (Yang et al. 2015). *Agrimonia pilosa* Ledeb (Rosaceae) reduces inflammation by suppressing inducible nitric oxide synthase (iNOS) and COX-2 and reactive oxygen species in LPS-activated RAW 264.7 macrophage cells (Jin et al. 2016). Protopine isolated from *Corydalis yanhusuo* W. T. Wang (Papaveraceae) improves renal function in acute renal injury mice induced by LPS. Protopine also alleviates the inflammatory response, inhibits inflammatory cytokines, and reduces apoptosis and necrosis via the Toll-like receptor 4 signalling pathway (Zhang et al. 2019). *Panax notoginseng* (Burk.) F.H. Chen (Araliaceae) inhibits LPS-stimulated NO-overproduction and iNOS overexpression via suppression of Toll-like receptor 2-mediated MAPK/NF-κB signalling pathways in RAW 264.7 macrophages (Ye et al. 2020).

**Table 1.** Different components in the formula of JPQCHSD.

Chinese name	Latin name	Used part	Traditional use	Voucher specimens
Dang shen	*Codonopsis pilosula* (Franch.) Nannf	Root	Qi-reinforcing	TCM – 201113 – JPQCHSD01
Danshen	*Salvia miltiorrhiza* Bge	Root	Blood-activating and Stasis-removing	TCM – 200930 – JPQCHSD02
Bai zhu	*Atractylodes macrocephala* Koidz	Root	Qi-reinforcing	TCM – 200816 – JPQCHSD03
Tu fulin	*Smilacis Glabrae* Rhizoma	Rhizome	Antipyretic detoxicate	TCM – 201022 – JPQCHSD04
Gan cao	*Glycyrrhiza uralensis* Fisch.	Root and rhizome	Qi-reinforcing	TCM – 201017 – JPQCHSD05
Mu xiang	*Aucklandia lappa* Decne.	Root	Qi-regulating	TCM – 200301 – JPQCHSD06
Huang lian	*Coptis chinensis* Franch.	Rhizome	Heat-Clearing and Dampness-drying	TCM – 201012 – JPQCHSD07
Hogn teng	*Sargentodoxa cuneata* (Oliv.) Rehd.et Wils.	Stem	Antipyretic detoxicate	TCM – 201004 – JPQCHSD08
Liu jinu	*Siphonostegia chinensis* Benth	Whole grass	Blood-activating and Stasis-removing	TCM – 200724 – JPQCHSD09
Xian hecao	*Agrimonia pilosa* Ledeb.	Aerial parts	Astringent haemostatic	TCM – 200906 – JPQCHSD10
Di yu	*Sanguisorba officinalis* L	Root	Blood-cooling and Haemostatic	TCM – 200802 – JPQCHSD11
Fu lin	*Poria cocos* (Schw.) Wolf.	Sclerotium	Diuretic and Dampness-excreting	TCM – 201028 – JPQCHSD12
Yan husuo	*Corydalis yanhusuo* W. T. Wang.	Rhizome	Qi-regulating	TCM – 201012 – JPQCHSD13
San qi	*Panax notoginseng* (Burk.) F.H. Chen	Root	Stasis-resolving and Haemostatic	TCM – 201108 – JPQCHSD14

**Table 2.** The components identified in the JPQCHSD.

Components	Concentration (μg/mL)
Danshensu	1004.683
Berberine hydrochloride	576.871
Chlorogenic acid	536.117
Astilbin	121.007
Kaempferol	111.972
Ginsenoside Rb1	101.407
Salidroside	101.270
Luteolin	92.269
Ginsenoside Rg1	81.019
Caffeic acid	55.903
Glycyrrhizin	44.377
Isoliquiritigenin	19.859
Notoginsenoside r1	19.241
Quercetin	18.228
Tetrahydropalmatine	17.818
Atractylenolide III	17.345
Atractylolide I	15.066
Rutin	5.414
Cryptotanshinone	5.197
Tanshinone I	1.525
Emodin	1.512
Costunolide	1.115
Protocatechuic acid	1.0
Tanshinone IIA	0.913

Thus, the ingredients in JPQCHSD possess anti-inflammatory effects in different diseases; however, few studies have focussed on the treatment of IBD with the components of JPQCHSD, and the potential protective effects of JPQCHSD extract on IBD have not been evaluated. Therefore, we preliminarily assessed these effects in a TNBS-induced rat model of IBD.

## Materials and methods

### Reagents

TNBS and pentobarbital were purchased from Sigma-Aldrich (St. Louis, MO). Superoxide dismutase (SOD), malondialdehyde (MDA), and myeloperoxidase (MPO) kits were obtained from Nanjing Jiancheng Biochemical Co., Ltd. (Nanjing, China). Immunoglobulin A (IgA) kits were obtained from Shanghai Kehua Bio-Engineering Co., Ltd., (Shanghai, China). Primers for reverse transcriptase-polymerase chain reaction (RT-PCR) were synthesized by Sangon Biotech, (Shanghai, China). Enzyme-linked immunosorbent assay (ELISA) kits for rat interleukin (IL)-1β, IL-6, and TNF-α were obtained from Beijing SINO-UK Institute of Biological Technology (Beijing, China). The RNAiso Plus kit was obtained from TaKaRa Technologies Co., Ltd. (Shiga, Japan). The cDNA synthesis kit (with gDNase) and SuperReal PreMix Plus (SYBR Green) were purchased from Tiangen Biotech (Beijing, China). Anti-NF-κBp65, streptavidin-peroxidase (SP), and 3,3′-diaminobenzidine kits were obtained from Bosen Biotechnology Co., Ltd. (Beijing, China).

### Animals

Male Sprague–Dawley rats weighing 180 ± 20 g were obtained from Beijing Weitong Lihua Research Centre for Experimental Animals and were maintained in a temperature-controlled room (25 ± 1 °C) on a 12 h light/dark cycle in the Animal Centre of the Beijing Hospital of Traditional Chinese Medicine (Capital Medical University Beijing, China). The study was conducted in accordance with guidelines for animal experimentation and the protocol was approved by the animal studies ethics committee of Beijing Hospital of Traditional Chinese Medicine, Capital Medical University with code number 2017070102.

### Preparation of JPQCHSD extract

JPQCHSD consists of the following (Table 1): *Codonopsis pilosula* (TCM – 201113 – JPQCHSD 01)*, Salvia miltiorrhiza* (TCM – 200930 – JPQCHSD 02)*, Atractylodes macrocephala* (TCM – 200816 – JPQCHSD 03)*, Smilacis glabrae* rhizoma (TCM – 201022 – JPQCHSD 04)*, Glycyrrhiza uralensis* (TCM – 201017 – JPQCHSD 05)*, Aucklandia lappa* (TCM – 200301 – JPQCHSD 06)*, Coptis chinensis* (TCM – 201012 – JPQCHSD 07)*, Sargentodoxa cuneata* (TCM – 201004 – JPQCHSD 08)*, Siphonostegia chinensis* (TCM – 200724 – JPQCHSD 09)*, Agrimonia pilosa* (TCM – 200906 – JPQCHSD10)*, Sanguisorba officinalis* (TCM – 200802 – JPQCHSD11)*, Poria cocos* (TCM – 201028 – JPQCHSD12)*, Corydalis yanhusuo* (TCM – 201012 – JPQCHSD13) and *Panax notoginseng* (TCM – 201108 – JPQCHSD 14) at a ratio of 3:3:3:6:3:3:3:6:10:5:10:3:3:1, with a total weight of 186 g (Table 1). All herbs were provided by Beijing Xinglin Pharmaceutical Co., Ltd. (Beijing, China) and met the standards specified in the Chinese Pharmacopoeia (2015 edition). Voucher specimens are stored in our Laboratory of Medicinal Plant and Pharmacognosy, Beijing Hospital of Traditional Chinese Medicine, Capital Medical University. Fourteen herbs were mixed and ground according to the proportions. JPQCHSD was extracted in distilled water at 100 °C for 2 h. The JPQCHSD solution was concentrated to 3.8 g crude herb/mL and stored at −20 °C until use.

### Ultra-high-performance liquid chromatography-tandem mass spectrometry (UPLC-MS/MS) analysis of JPQCHSD

UPLC-MS/MS was used to analyze the components of the JPQCHSD formula. The collision voltages of the positive and negative ion sources were 3.0 and 2.2 kV, respectively; the temperature of the heated vaporizer was 300 °C; and nitrogen was used as the sheath, auxiliary, and collision gas. Waters Masslynx 4.1 and UNIFI Scientific Information Systems (Milford, MA) were used to collect and analyze the data. The Waters Acquity UPLC H-Class system was used to analyze the components of JPQCHSD with a chromatographic column (UPLC HSS T3 column (2.1 × 100 mm, 1.8 mm). The mobile phase consisted of A (water: 0.1% formic acid, v/v) and B (acetonitrile) and was run at a flow rate of 0.4 mL/min with a column temperature of 55 °C.

### Animal experimental protocol

During adaptive breeding, the rats had free access to food and water, and 40 rats were randomly divided into five groups (*n* = 8). The method used to establish the IBD rat model has been described previously (Karaboga et al. 2017; Chamanara et al. 2019; Cota et al. 2019). After fasting the rats overnight, each rat was administered intraperitoneal anaesthesia of 10% chloral hydrate and a silicone tube lubricated with paroline was inserted approximately 8 cm into the colon through the anus; except the rats of control group, 150 mg/kg TNBS (dissolved in 1 mL of 50% ethanol) was administered through the tube. After the TNBS enema, the anus was squeezed, the tail was lifted, and the rat was held upside down for 3 min to prevent TNBS leakage. While, 50% ethanol was instilled in the intestines of control rats without TNBS. The groups were: (1) Control, administration of normal saline orally; (2) Model, administration of normal saline orally; (3) JPQCHSD 9.5 g/kg; (4) JPQCHSD 19 g/kg; (5) JPQCHSD 38 g/kg. The rats were administered 9.5, 19, and 38 g/kg of JPQCHSD water extract by oral gavage once per day for 12 days. On day 13, the rats were sacrificed by excessive inhalation of ether.

### Sample collection

The abdomen was opened quickly, and the colon was removed. The colon was cut open with surgical scissors, washed gently with normal saline, and one piece of the colon was placed in 4% paraformaldehyde for subsequent pathological section analysis. The remaining colon was frozen in liquid nitrogen and stored at −80 °C for subsequent analysis.

### Histological assessment of IBD

Haematoxylin and eosin (H&E) staining was conducted to analyze the morphological characteristics of the rat colon tissue. Samples collected from the resected colon were fixed in 10% buffered formalin, dehydrated in different concentrations of ethanol, and embedded in paraffin. Next, 5 μm thick sections were cut and graded using a 0–3 scale as previously described (Strus et al. 2015) for histological examination: 0: colon showed no inflammatory response; 1: small number of infiltrating cells in the lamina propria (significant acute inflammation); 2: large number of infiltrating neutrophils (moderate acute inflammatory injury); and 3: ulcerated and granulated tissue (chronic type injury).

### Assessment of IBD

During the experiment, the weight of the rats, stool appearance and consistency, and gross rectal bleeding were constantly monitored. IBD severity and disease activity of each rat were assessed using the disease activity index (DAI) score as previously described (Murthy et al. 1993) using a 0–4 point system focussed on the major elements of stool, faecal occult blood, and weight loss. Rat stool samples were collected and evaluated daily and faecal occult blood test papers (Beijing Reagan Biotechnology Co., Ltd., Beijing, China) were used to test for faecal occult blood following the manufacturer’s protocol. For body weight, points were allocated as follows: 0: no weight loss; 1: 1–5% weight loss; 2: 5–10% weight loss; 3: 10–15% weight loss; and 4: >15% weight loss. The stool consistency was described as follows: 0: normal; 1: slightly loose; 2: loose; and 3: diarrhoea. The DAI value was calculated according to the following formula: (weight reduction score + stool consistency score + bleeding score)/3 (Hamamoto et al. 1999; Silvia Melgar and Erik 2005).

### Determination of SOD activity and MDA level in colon

Colon tissue samples stored at −80 °C were ground into tissue homogenates and the supernatant was collected by spinning at 15,000*g* for 10 min. The SOD and MDA contents in the supernatant were determined using corresponding kits. Based on the production of superoxide anion radical, the activity of SOD was determined by the xanthine oxidase method. The absorbance was determined at 550 nm by spectrophotometry, and SOD activity were expressed in units per milligram of protein (U/mg protein). MDA levels in the colonic samples were tested by the method of thiobarbituric acid (TBA). The absorbance was determined at 532 nm by spectrophotometry and MDA levels were expressed in nanomols per milligram of protein (nmol/mg protein).

### Detection of MPO activity in Colon

The excised colon tissue samples were homogenized in 0.1 M phosphate buffer (pH 7.4) and centrifuged at 15,000 *g* for 10 min, and then MPO activity was determined using an MPO test kit. Spectrophotometry was used to detect the enzyme activity. MPO activity was determined by measuring the absorbance of change at 460 nm caused by MPO-catalyzed oxidation of 3, 3′-dimethoxybenzidine. MPO activity was expressed in units per milligram of protein (U/mg protein) and an enzyme activity unit was defined as 1 μmol hydrogen peroxide decomposed per gram of colon tissue at 37 °C.

### Detection of immunoglobulin A (IgA) concentration in serum

Blood samples were centrifuged at 3000 rpm and 4 °C for 15 min to obtain the serum and then IgA concentration was determined using an IgA test kit. IgA in serum combines with its corresponding specific antibody to form antigen antibody complex, and produces a certain turbidity, which is directly proportional to the content of IgA in the sample. The absorbance was determined at 340 nm and the sample concentration was calculated.

### ELISA

The serum levels of TNF-α, IL-1β, and IL-6 were detected using ELISA kits according to the manufacturers’ protocols. Briefly, purified biotinylated antibody was used to coat the microporous plate to generate solid-phase antibody and colon tissue lysate and horseradish peroxidase-labelled avidin were added. After thorough washing, 3,3′,5,5′-tetramethylbenzidine was added for colour development. The absorbance was determined at 450 nm and the sample concentration was calculated.

### RT-PCR

RNA was extracted from the resected colon tissue samples preserved at −80 °C using a kit according to the manufacturer’s protocol. The following PCR primers were synthesized. NF-κB: forward, 5′-GTCATCAGGAAGAGGTTTGGCT-3′′ and reverse, 5′-TGATAAGCTTAGCCCTTGCAGC-3′′ (Arab et al. 2014) and glyceraldehyde 3-phosphate dehydrogenase (GAPDH): forward, 5′-TGACTCTACCCACGGCAAGTTCAA-3′′ and reverse, 5′-ACGACATACTCAGCACCAGCATCA-3′′ (Sangon Biotech, Shanghai, China). cDNA was synthesized using a RevertAid first-strand DNA synthesis kit (Tiangen Biotech, Beijing, China). ABI-7500 Real-time quantitative PCR thermal cycl and a real-time PCR system kit (Tiangen Biotech, Beijing, China) were used to detect Thermal cycler gene expression according to the manufacturer’s instructions and cycle threshold (CT) values were calculated by normalizing CT to GAPDH expression to present target gene (2^−ΔΔCT^) expression. The SYBR Green real-time PCR cycling protocol used was as follows: DNA denaturation at 95 °C for 3 min, followed by 40 cycles at 95 °C for 5 s and 60 °C for 33 s.

### Immunohistochemistry

The 5 μm thick tissue sections were deparaffinized and rehydrated using xylene and ethanol, followed by incubation in 3% aqueous hydrogen peroxide to inhibit endogenous peroxidase activity and goat serum (Bosen Biotechnology Co., Ltd., Beijing, China) to block non-specific staining. The sections were then incubated with primary antibodies against NF-κB (1:100) in diluent buffer (Bosen Biotechnology Co., Ltd.) at 37 °C for 1 h, followed by a biotinylated secondary antibody and horseradish enzyme-labelled Streptomyces ovalbumin working solution (SP kit; Bosen Biotechnology Co., Ltd.) at 37 °C for 30 min each. Finally, 3,3′-diaminobenzidine solution was applied, the sections were counterstained with Mayer’s haematoxylin and observed under a light microscope (Zeiss GmbH, Jena, Germany). ImageJ software (National Institutes of Mental Health, Bethesda, MD) was used to analyze the images. The cytoplasm and nuclei of cells labelled as brown were considered as positive.

### Statistical analysis

The statistical analysis results are expressed as the means ± standard deviation (x̄ ± s). The results were analyzed by one-way analysis of variance using SPSS version 17.0 software (SPSS, Inc., Chicago, IL). Differences were considered statistically significant when *p* < 0.05.

## Results

### Identification of major components of JPQCHSD formula

**Figure 1.**
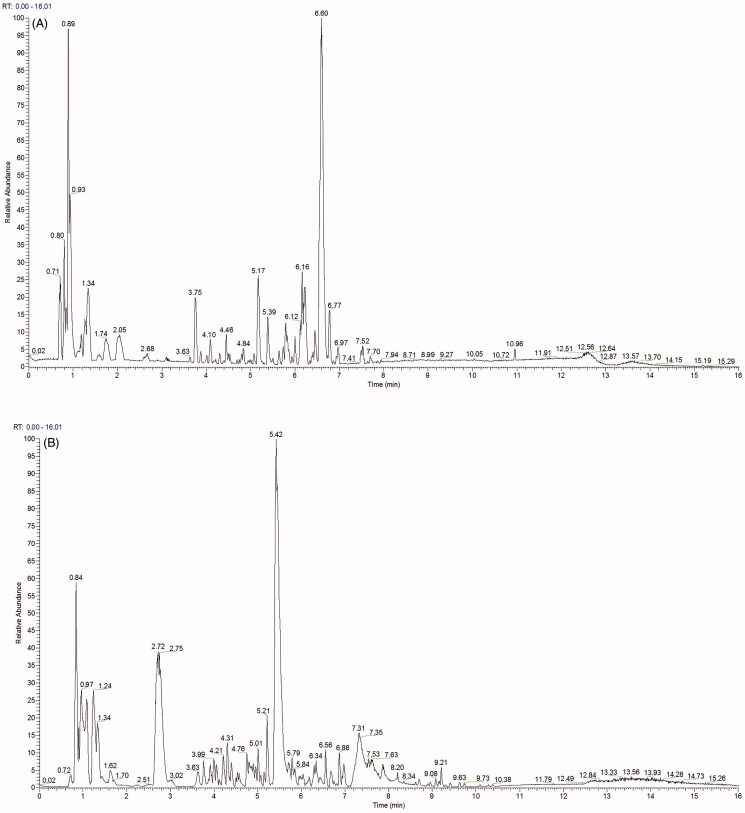
Analysis of the main components of Jian Pi Qing Chang Hua Shi decoction (JPQCHSD). Ultra-high-performance liquid chromatography-tandem mass spectrometry (UPLC-MS/MS) was used to identify the components of JPQCHSD and Masslynx 4.1 software was used to process and analyze the data. (A) Positive and (B) negative ion chromatograms of JPQCHSD are shown.

The components of JPQCHSD were evaluated by UPLC-MS/MS. The total ion chromatogram of the JPQCHSD formula is shown in Figure 1. According to the Chinese Pharmacopoeia (2015), the components identified in JPQCHSD were listed as follows: salidroside, danshensu, astilbin, notoginsenoside r1, ginsenoside Rg1, emodin, rutin, tetrahydropalmatine, isoliquiritigenin, tanshinone IIA, atractylenolide III, tanshinone I, costunolide, atractylolide I, kaempferol, glycyrrhizin, berberine hydrochloride, cryptotanshinone, quercetin, luteolin, chlorogenic acid, protocatechuic acid, caffeic acid, ginsenoside Rb1 (Table 2).

### Protective effects of JPQCHSD on TNBS-induced IBD

**Figure 2.**
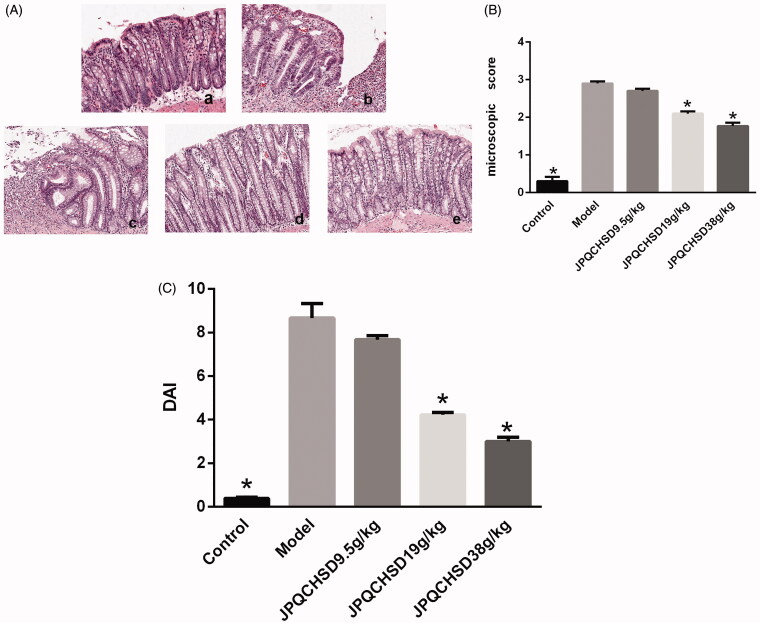
Effect of Jian Pi Qing Chang Hua Shi decoction (JPQCHSD) on histopathological changes in 2-4-6-trinitrobenzene sulphonic acid (TNBS)-induced inflammatory bowel disease (IBD) model. (A) Representative images of haematoxylin and eosin (H&E) staining: a. control; b. model; and c-e. JPQCHSD 9.5 g/kg, JPQCHSD 19 g/kg, and JPQCHSD 38 g/kg. (B) Microscopy score was used for histological evaluation. (C) Effect of JPQCHSD on disease activity index (DAI). JPQCHSD 19 g/kg and JPQCHSD 38 g/kg, but not JPQCHSD 9.5 g/kg, significantly decreased pathological damage in IBD and reduced increased DAI and microscopy scores; ^★^*p* < 0.05 as compared to the model group. Data are shown as the mean ± standard deviation. Control, no treatment; Model, TNBS-induced IBD rats.

H&E staining (Figure 2(A,B)) to evaluate histological characteristics showed that colon tissue samples from the control group had normal, typical structures without hyperaemia, oedoema, erosion, or ulcers, whereas those from the TNBS-induced model group exhibited considerable inflammatory cell infiltration, mucosal oedoema, and thickening of the colon wall. The JPQCHSD (19 g/kg) and JPQCHSD (38 g/kg)-treated groups exhibited significant less inflammatory cell infiltration and 0 ulceration than the model group. The microscopic score of colon mucosal injury was consistent with that of H&E staining. TNBS induced severe inflammatory damage to the model group, whereas this effect was inhibited by treatment with JPQCHSD 19 g/kg and JPQCHSD 38 g/kg. IBD-induced rats showed significant weight loss, diarrhoea, and bloody stool. The DAI (Figure 2(C)) of TNBS-induced IBD rats was significantly higher (*p* < 0.05) than that of control rats. However, administration of different doses of JPQCHSD (19 and 38 g/kg) significantly decreased the DAI of IBD rats (*p* < 0.05) compared to the control group, which was accompanied by notable alleviation of diarrhoea and faecal haemorrhage (Figure 2). The ED_50_ value for the disease activity index was 26.93 g/kg.

### Effects of JPQCHSD on MDA level and SOD activity in colon

**Figure 3.**
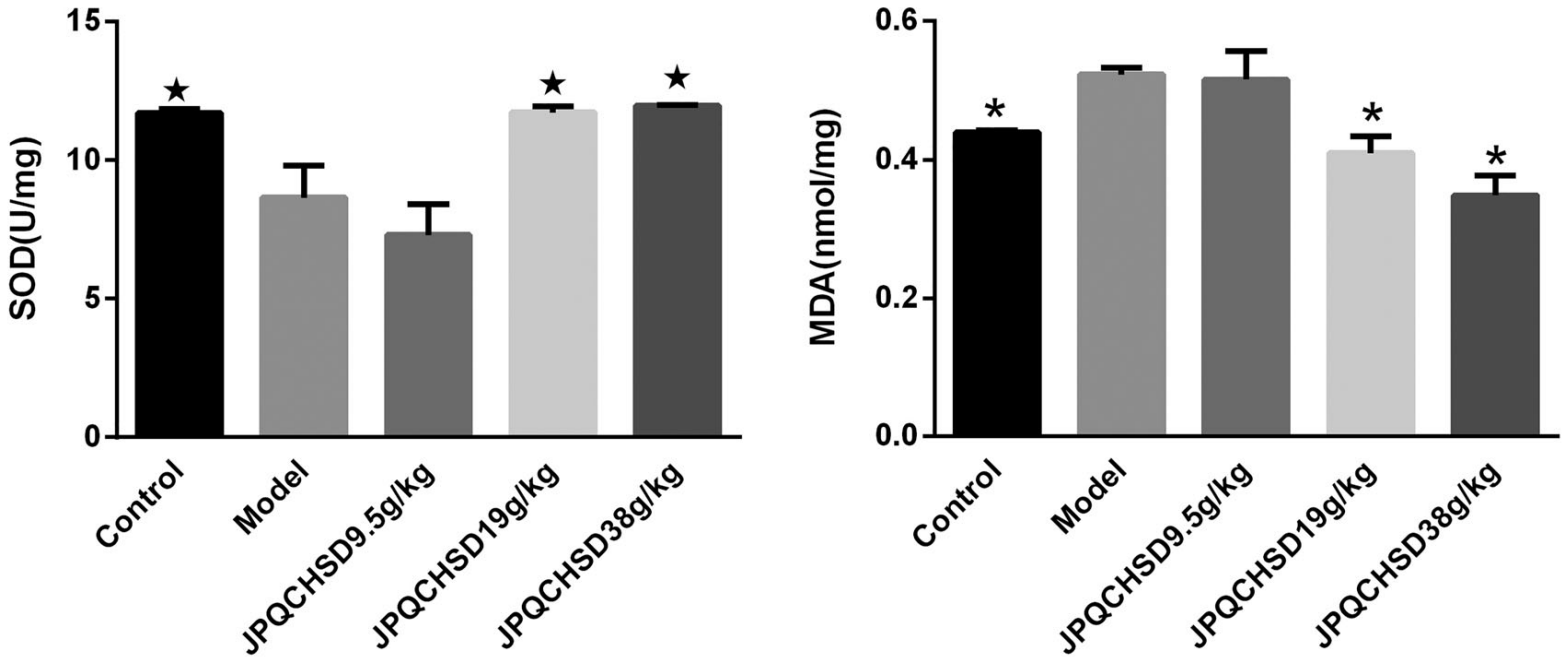
Effects of Jian Pi Qing Chang Hua Shi decoction (JPQCHSD) on levels of malondialdehyde (MDA) and superoxide dismutase (SOD) activity in colon in 2,4,6-trinitrobenzene sulphonic acid (TNBS)-induced inflammatory bowel disease (IBD) rats. MDA and SOD levels were measured. ^★^*p* < 0.05 and *p < 0.05 as compared to the model group. Data are shown as the mean ± standard deviation. Control, no treatment; Model, TNBS-induced IBD rats; JPQCHSD 9.5 g/kg, JPQCHSD 19 g/kg, and JPQCHSD 38 g/kg, respectively.

The MDA level in the model group was significantly higher than that in the control group (Figure 3). Furthermore, both the JPQCHSD 19 g/kg and JPQCHSD 38 g/kg groups showed significantly lower MDA levels than the model group. SOD activity in the colon was significantly lower in TNBS-induced IBD rats than in control rats, whereas JPQCHSD 19 g/kg and JPQCHSD 38 g/kg, but not JPQCHSD 9.5 g/kg, significantly increased these levels (Figure 3). The ED_50_ values for the increased SOD and decreased MDA were 48.98 and 53.2 g/kg, respectively.

### Effects of JPQCHSD on MPO activity in the colon

**Figure 4.**
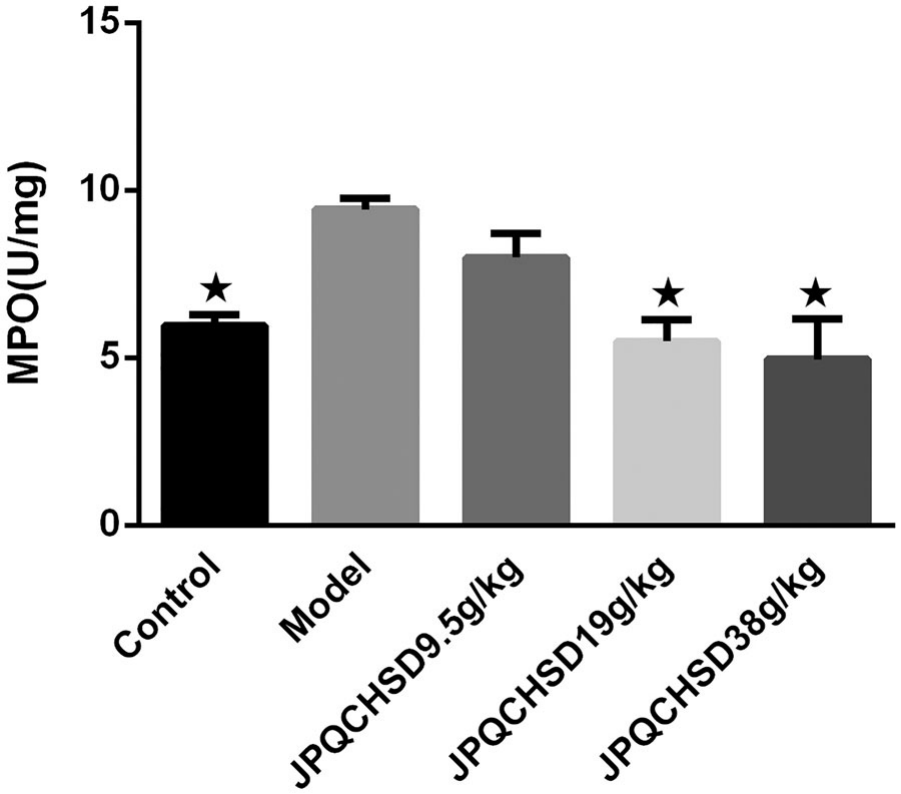
Detection of colonic myeloperoxidase (MPO) activity. MPO activity was significantly higher in 2,4,6-trinitrobenzene sulphonic acid (TNBS)-induced inflammatory bowel disease (IBD) rat model than in control rats. Both JPQCHSD 19 g/kg and JPQCHSD 38 g/kg groups showed low MPO activities. ^★^*p* < 0.05 as compared to model group. Data are shown as the mean ± standard deviation. Control, no treatment; Model, TNBS-induced IBD rats.

MPO activity was significantly higher in the TNBS-induced model group than in the control group (*p* < 0.05). JPQCHSD 19 g/kg and JPQCHSD 38 g/kg significantly decreased MPO activity in the colon (*p* < 0.05) (ED_50_ values 37.38 g/kg). Although the MPO level in the JPQCHSD 9.5 g/kg group was decreased, the result was not significantly different from that in the model group (Figure 4).

### Effects of JPQCHSD on concentration of serum IgA

**Figure 5.**
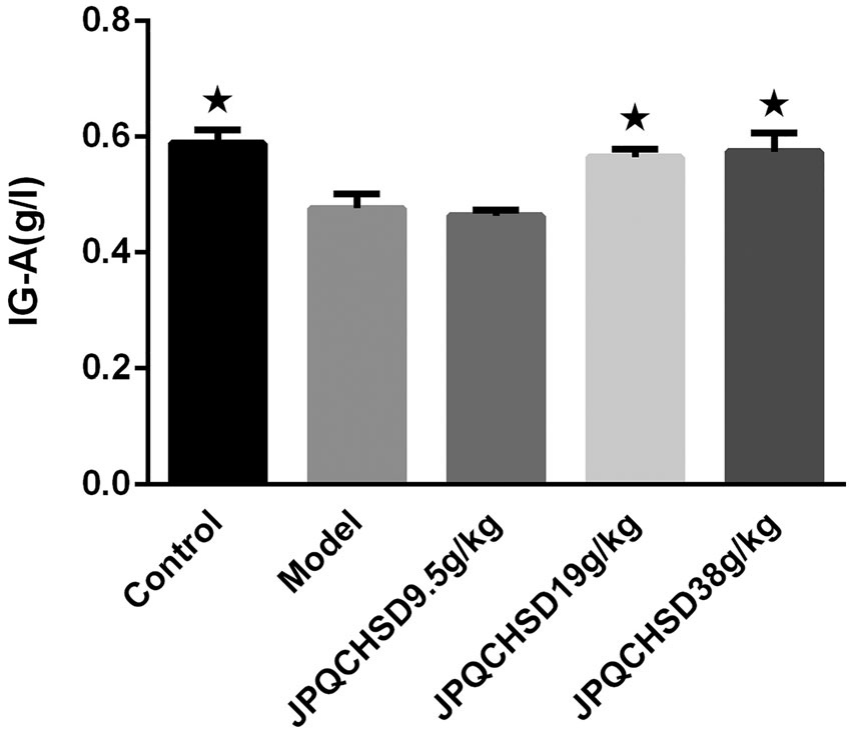
Effects of JPQCHSD on concentration of serum immunoglobulin A (IgA). Compared with the normal group, the content of IgA in the serum of the model was decreased significantly (*p* < 0.05). Compared with the model group, the IgA content in JPQCHSD 19 g/kg and JPQCHSD 38 g/kg groups were increased (*p* < 0.05), while the IgA level in the JPQCHSD 9.5 g/kg group was not significantly increased (*p* > 0.05). ^★^*p* < 0.05 as compared to the model group. Data are shown as the mean ± standard deviation. Control, no treatment; Model, TNBS-induced IBD rats.

Compared with the normal group, the content of IgA in the serum of the model was decreased significantly (*p* < 0.05). Compared with the model group, the IgA content in JPQCHSD 19 g/kg and JPQCHSD 38 g/kg groups were increased (*p* < 0.05). The ED_50_ values for the increased IgA were 56.3 g/kg. The IgA level in the JPQCHSD 9.5 g/kg group was not significantly increased (*p* > 0.05) (Figure 5).

### Effects of JPQCHSD on protein expression of IL-1β, IL-6, and TNF-α

**Figure 6.**
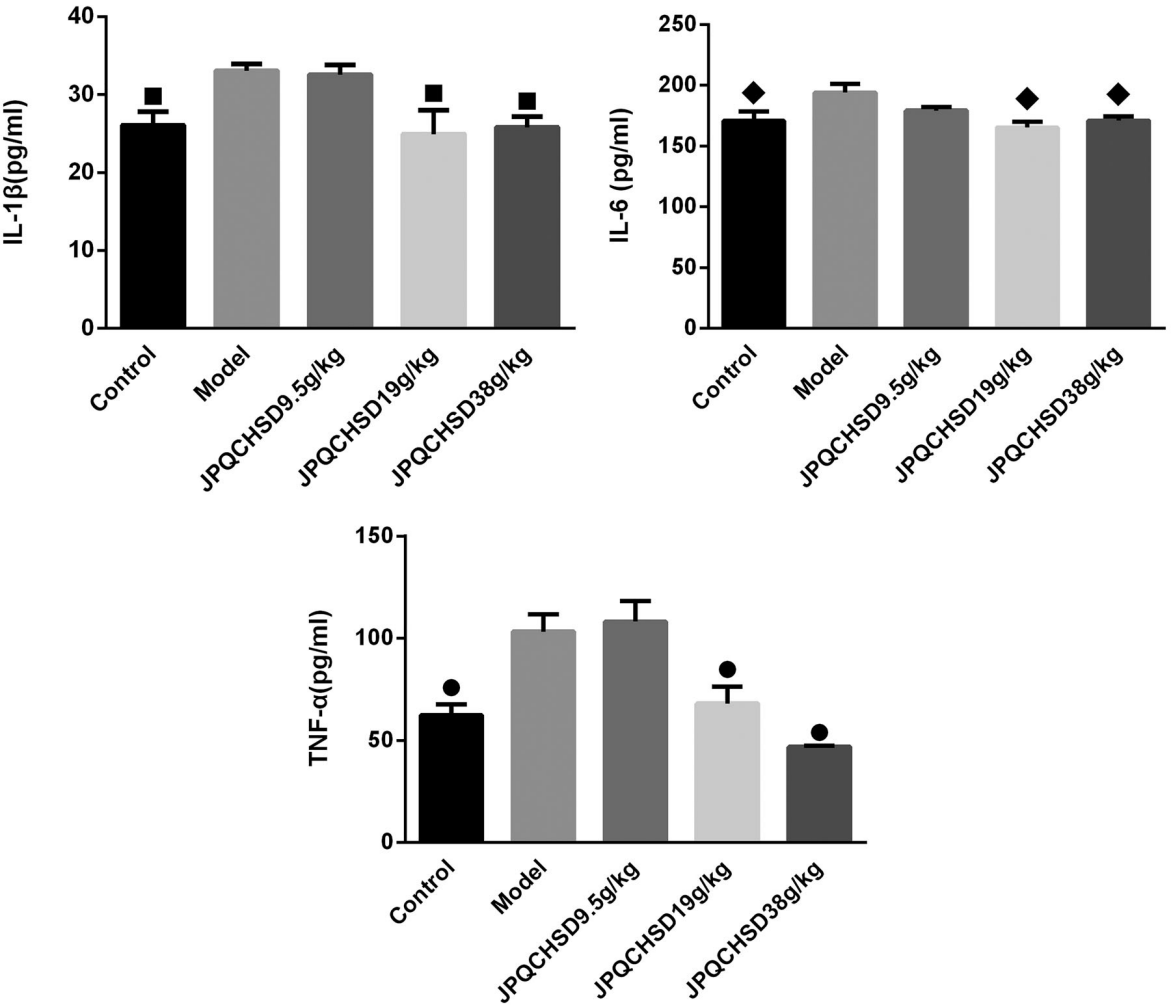
Serum interleukin (IL)-1β, IL-6, and tumour necrosis factor (TNF)-α levels of the five groups. ^■^*p* < 0.05, ^◆^*p* < 0.05, and ^●^*p* < 0.05 as compared to the model group. Data are shown as the mean ± standard deviation. Control, administered 0.9% saline; model, 2-4-6-trinitrobenzene sulphonic acid (TNBS)-induced inflammatory bowel disease (IBD) rats; JPQCHSD 9.5 g/kg, JPQCHSD 19 g/kg, and JPQCHSD 38 g/kg, respectively.

The effect of JPQCHSD on inflammatory cytokines was examined by ELISA; TNF-α, IL-6, and IL-1β protein expression levels were significantly higher in the model group than in the control group (Figure 5). Although there was no significant difference in the serum levels of these cytokines between the JPQCHSD 9.5 g/kg and model groups (Figure 6), JPQCHSD 19 g/kg and JPQCHSD 38 g/kg treatment reduced IL-1β, IL-6, and TNF-α protein expression levels and ED_50_ values for inhibition of TNF-α, IL-1β, and IL-6 were 32.66, 75.72, and 162.06 g/kg, respectively.

### Effects of JPQCHSD on mRNA and protein expression of NF-κB

**Figure 7.**
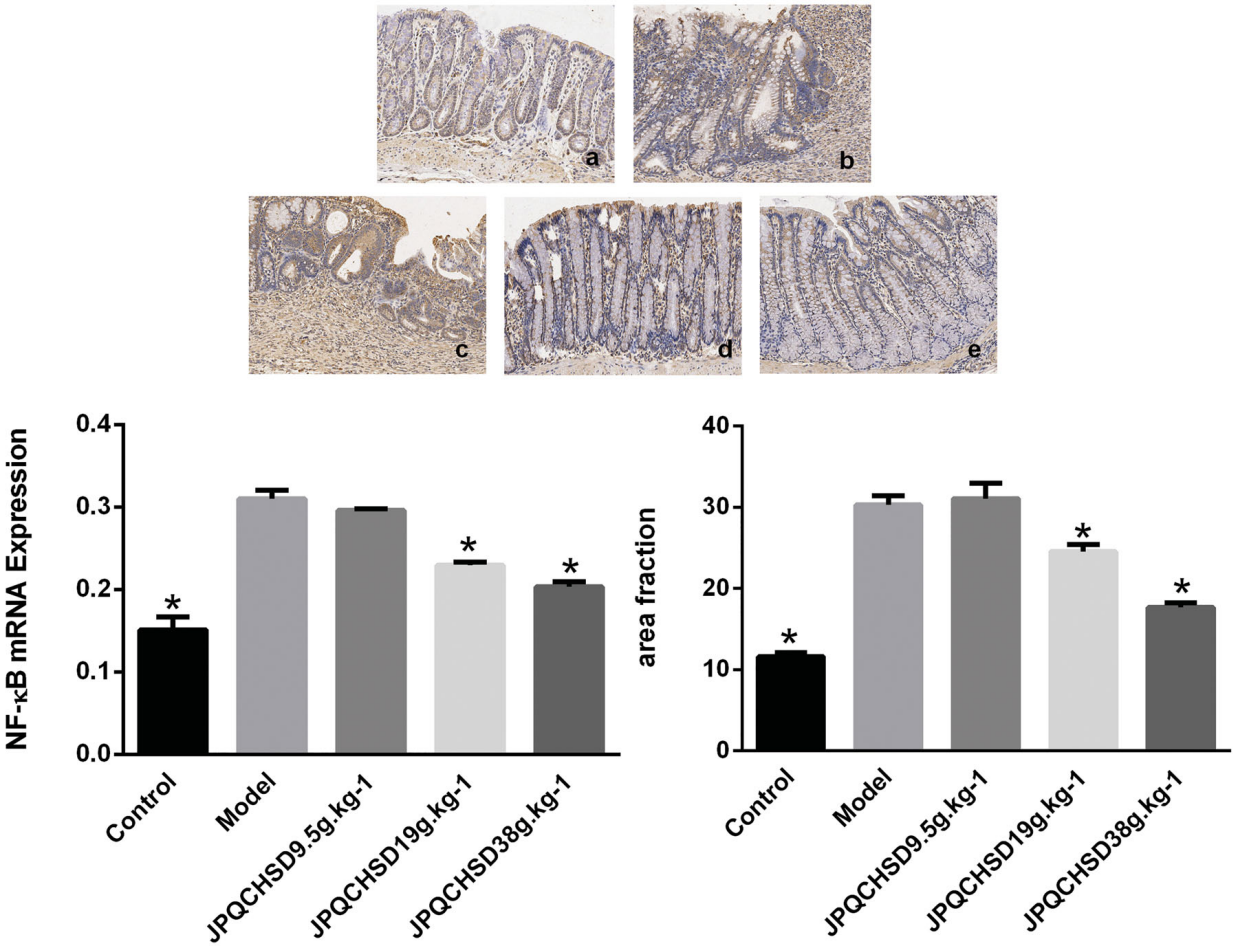
Nuclear factor (NF)-κB mRNA and protein expression levels in colon tissue of five groups. *p < 0.05 as compared to the model group. Data are shown as the mean ± standard deviation. a. Control, no treatment; b. Model, 2,4,6-trinitrobenzene sulphonic acid (TNBS)-induced inflammatory bowel disease (IBD) rats; c–e. JPQCHSD 9.5 g/kg, JPQCHSD 19 g/kg, and JPQCHSD 38 g/kg, respectively.

The NF-κB p65 mRNA and protein expression levels in the colon tissue samples were examined by real-time PCR and immunohistochemistry. NF-κB p65 mRNA expression was significantly increased in TNBS induced IBD rats. Compared with the model group induced by TNBS, 19 and 38 g/kg JPQCHSD could significantly decrease NF-κB p65 mRNA expression (*p* < 0.05), however the 9.5 g/kg JPQCHSD failed to inhibit NF-κB expression. While immunohistochemistry staining results also showed that TNBS-induced IBD rats treated with JPQCHSD 19 and 38 g/kg, but not JPQCHSD 9.5 g/kg, showed significantly lower NF-κB p65 protein expression levels compared to TNBS-induced model rats (*p* < 0.05, Figure 7).

## Discussion

In this study, we performed preliminary evaluation of the protective effects of JPQCHSD extract on IBD in an experimental rat model. IBD, a chronic progressive inflammatory bowel disease, is one of the main risk factors for colon cancer and its development is associated with cellular immunity, oxidative stress, and autoimmunity. Immune dysfunction plays an important role in the pathogenesis of IBD (Szczeklik et al. 2019). Oxygen-free radicals are closely related to local inflammation in IBD. Oxygen-free radicals are a group of oxygen-containing groups that are active in nature and have a strong oxidative effect, which causes great damage to living organisms. Excessive oxygen-free radicals cause lipid peroxidation of unsaturated fatty acids, resulting in increased levels of lipid peroxides that decompose to products such as MDA. MDA further aggravates tissue damage and leads to protein denaturation, cross-linking DNA, and cell membrane structural damage. Furthermore, tissues release numerous inflammatory mediators, which aggravate tissue damage. MDA also inactivates hormones and enzymes in the body, leading to immune function decline and the destruction of intracellular nucleic acid structure of tissues and, ultimately, a variety of diseases. MDA levels in patients with IBD were significantly increased, thus, MDA may be an effective index for the severity of IBD (Nikkhah-Bodaghi et al. 2019; Oliveira et al. 2019; Wang et al. 2019; Zhao et al. 2019).

To maintain the physiological balance, the body contains an antioxidant system consisting of enzymes such as SOD, which can effectively scavenge oxygen-free radicals and inhibit lipid peroxidation in the colon tissue (Babbs 1992). The SOD content indirectly reflects oxygen-free radical scavenging ability. The activity of SOD in IBD is significantly lower than that in controls without IBD; thus, SOD may be an effective treatment target for IBD (Kaulmann and Bohn 2016; Wang et al. 2017). In our study, the contents of SOD and MDA in the colonic mucosa of IBD rats were lower and higher, respectively, than in control rats.

A decreased SOD content indicates that the oxygen-free radical scavenging ability is decreased, whereas an increased MDA content indicates increased free radical production, suggesting that an imbalance in oxidative metabolism occurred in IBD rats. Treatment with JPQCHSD 19 and 38 g/kg increased SOD and decreased MDA, indicating that JPQCHSD restored the scavenging ability at medium and high doses. In the treatment of IBD, JPQCHSD may regulate the level of oxygen-free radicals. JPQCHSD 9.5 g/kg failed to restore the scavenging ability, likely because of the weak pharmacological effects of the low dose. *Codonopsis pilosula, Salvia miltiorrhiza* and *Sargentodoxa cuneata* have similar effects by inhibiting MDA levels and increasing SOD activities in different diseases (Xiao et al. 2005; Wang et al. 2011; Zhang et al. 2017). Their anti-inflammatory effects are similar to those of JPQCHSD but their dose are higher than those of JPQCHSD 19 g/kg and JPQCHSD 38 g/kg.

MPO is mainly secreted by neutrophils; thus, changes in MPO activity indirectly reflect the level of neutrophil infiltration and inflammation in tissues (Masoodi et al. 2011; Muthas et al. 2017). Clinical studies have shown that intestinal mucosal MPO activity can be used as an indicator of the severity of IBD (Hansberry et al. 2017; Colombo et al. 2018) and, in the faeces, as a marker of IBD activity. Animal experiments have shown that MPO activity is significantly increased in colonic inflammation (Yukitake et al. 2011), whereas treatment with Guchangzhixie-Pill alleviates this inflammation and decreases MPO activity (Yang et al. 2017). Thus, MPO activity can be used as a quantitative indicator of the degree of colonic inflammation (Yang et al. 2017).

In our study, the MPO activity in the model group was higher than that in the control group, indicating that neutrophil infiltration occurred in IBD rats. MPO utilizes hydrogen peroxide and chloride ions to produce hypochlorite and form oxidizing free radicals, which damage intestinal cells. Treatment with JPQCHSD 19 g/kg and JPQCHSD 38 g/kg decreased MPO, indicating that JPQCHSD inhibited neutrophil infiltration and regulated immune cells. The lack of a significant difference between the effect of JPQCHSD 9.5 g/kg and the model group suggests that the extract exerts a weak pharmacological effect at a low dose.

When inflammatory cell infiltration occurs, IBD is induced in the colonic tissue and TNF-α and IL-1β are released by inflammatory cells. TNF-α, an important mediator of biological immune defences and homeostasis, is closely related to IBD injury (Perrier and Rutgeerts 2011). TNF-α induces inflammatory cell aggregation by binding to high-affinity receptors on vascular endothelial cells, thereby upregulating the expression of IL-6 and adhesion molecules (Akanda et al. 2018). IL-1β is mainly produced by monocytes and macrophages. When patients with IBD have crypt inflammation, the content of IL-1β in intestinal fluids increases significantly. IL-1β interacts with antigens to activate CD4+ T cells, express IL-2R, and promote the activation and growth of B cells. IL-1β promotes the expression of antigen-presenting cells, such as monocytes and macrophages, and attracts neutrophils to aggregate and release inflammatory transmitters (Evgenikos et al. 2002). IL-6 is a cytokine that mediates the pathogenesis of IBD. The imbalance in pro-inflammatory cytokines, including TNF-α and IL-1β, plays a key role in the signalling cascade of the inflammatory pathway. TNF-α and IL-1β are the therapeutic targets of IBD therapy (Pedersen et al. 2014; Wallace et al. 2014).

The level of serum IL-6 in patients with IBD is significantly increased (Street et al. 2004; Traish et al. 2018) and changes in serum IL-6 are related to lesion activity. Increased IL-6 levels in IBD rats are related to the degree of colonic inflammation and can be used as an index to monitor inflammation (Tatiya-Aphiradee et al. 2018; Traish et al. 2018). Active IBD is associated with high levels inflammatory cytokines in peripheral blood. Serum IL-1β, IL-6, and TNF-α were elevated in the active phase of IBD patients’ and the IBD patient’s disease activity had strong correlations with the IL-1β, IL-6, and TNF-α (Mahmud et al. 1995; Niederau et al. 1997; Bitton et al. 2001; Hanai et al. 2006; Zwolinska-Wcislo et al. 2009; Bamba et al. 2014; Komine-Aizawa et al. 2015; Wu P et al. 2015).

NF-κB is an important inflammatory mediator, and factors such as endotoxin, lipopolysaccharide, phospholipase A, lysophosphatidylcholine, oxidative stress metabolites, viruses, and ischemia-reperfusion can stimulate the NF-κB signalling pathway. NF-κB regulates target gene transcription and promotes the generation of numerous inflammatory cytokines, growth factors, and acute phase proteins including TNF-α and IL-1β. NF-κB is highly expressed in IBD, where it plays an important role (Colombo et al. 2018) and induces the expression of TNF-α, IL-1β, and IL-6 (Fang et al. 2019). NF-κB inhibitors significantly reduce the infiltration of inflammatory cells and repress the expression of TNF-α, IL-6, and other inflammatory cytokines. NF-κB inhibitors effectively reduce the inflammatory response of colonic tissue, supporting the pivotal role of NF-κB in the occurrence and development of IBD (Samman et al. 2018; Güvenç et al. 2019).

This study showed that the expression level of NF-κB in colonic tissue of the model group was significantly higher than that in the normal control group and NF-κB, TNF-α, IL-1β, and IL-6 levels corresponded to the severity of colonic inflammatory damage. Thus, cytokines play a role in the occurrence and development of IBD. Additionally, increased expression of p65-NF-κB transcription of model group rats was significantly higher than that in the other groups. Higher transcription expression of NF-κB indicates higher expression levels of TNF-α, IL-1β, and IL-6. During the development of IBD, NF-κB transcription is increased, mainly because of lipid peroxidation. Therefore, an effective strategy for inhibiting NF-κB transcription is to reduce lipid peroxidation and block the NF-κB pathway, which would be useful for treating IBD (Chamanara et al. 2019). This study showed that JPQCHSD significantly reduced the expression of NF-κB in IBD rats and downregulated the expression of TNF-α, IL-1β, and IL-6. JPQCHSD thereby alleviated neutrophil infiltration and the inflammatory response in IBD.

This is the first study to evaluate the mechanism of JPQCHSD in the treatment of IBD. We investigated JPQCHSD at low, medium, and high doses to evaluate the efficacy of JPQCHSD for treating IBD and understand the anti-inflammatory mechanism. However, in this study, we did not compare the whole prescription and single traditional Chinese component herbs to verify whether JPQCHSD has stronger anti-inflammation effects than each individual ingredient. Lancemaside A; atractylenolide III*a*; berberine; costunolide; liriodendrin and notoginsenoside R1 may all decrease IL-1β, IL-6 and TNF-α expression by repressing NF-κB signal transducer in IBD mice or rats (Joh et al. 2010; Zhang et al. 2015; Han et al. 2017; Zhang et al. 2017; Li et al. 2019). Treatment with JPQCHSD 19 g/kg and JPQCHSD 38 g/kg also suppressed TNF-α, IL-6, and IL-1β levels in IBD rats. JPQCHSD 9.5 g/kg may exert a weak suppressive effect on the release of inflammatory cytokines. But JPQCHSD composed of a variety of traditional Chinese Medicine, has a multi-target advantage.

Previous studies have reported a reduction in IgA levels in patients with IBD (Ojuawo et al. 1997). IgA is the most abundant Igs in humans and the first line of specific immunological defence against environmental antigens (Reyna-Garfias et al. 2010). Compared with the control group, the level of serum IgA in the IBD mouse decreased (Gong et al. 2019), which is in agreement with our study. The IgA content in the serum of IBD rats is significantly decreased, whereas after JPQCHSD treatment, the IgA content is increased. Its mechanism is most likely related to enhancement of intestinal immunity. Combining with our data, JPQCHSD may not only inhibit inflammation but also regulate the immune system to treat IBD.

To clarify the exact therapeutic effect of JPQCHSD in the treatment of IBD, we will gradually subtract one or several herbs from JPQCHSD and split them into single medicines or drug groups to observe the changes in therapeutic effects. These important factors will also be evaluated in our follow-up research.

## Conclusions

**Figure 8.**
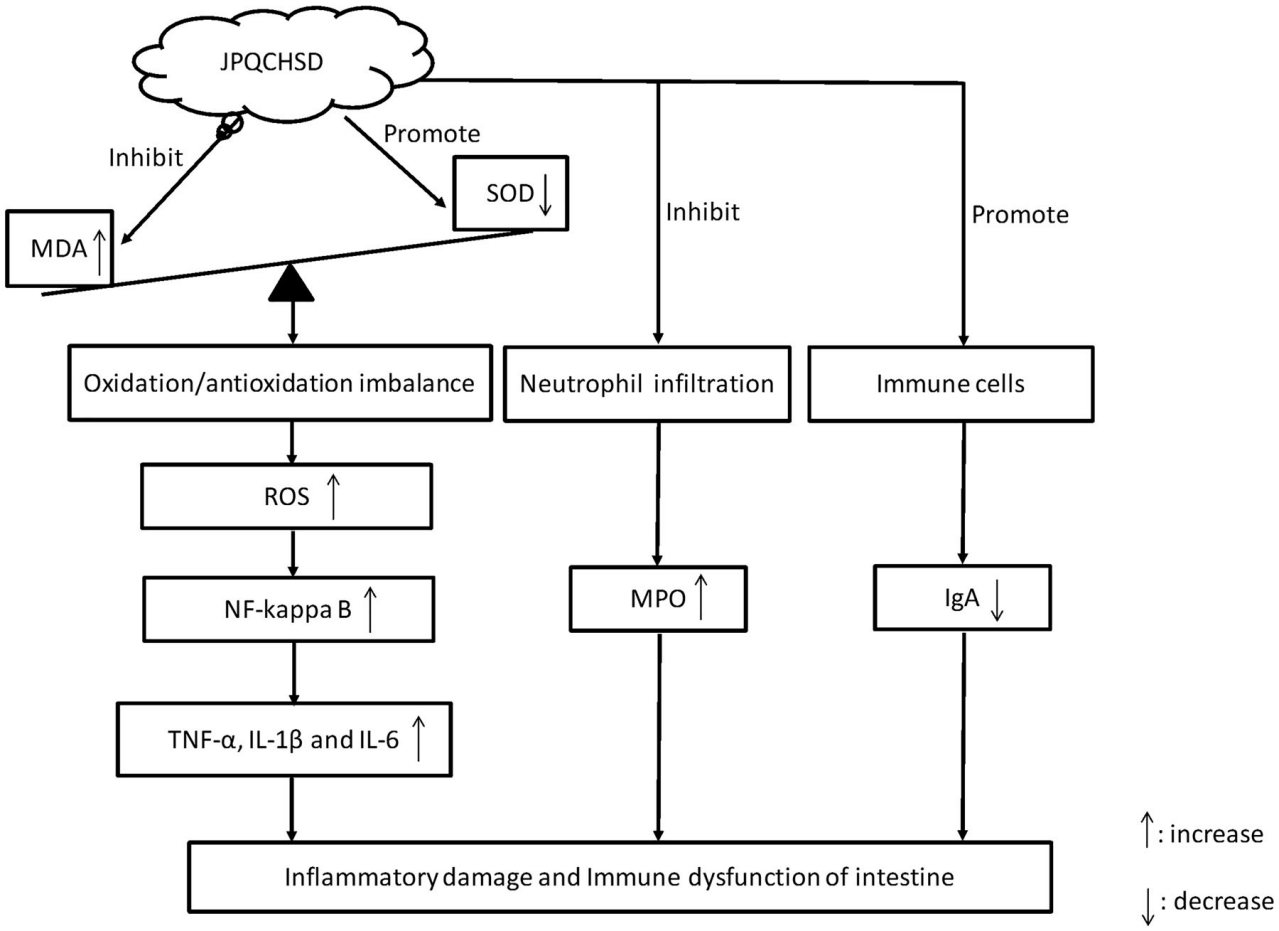
Jian Pi Qing Chang Hua Shi decoction (JPQCHSD) improved inflammatory damage to intestinal cells by increasing superoxide dismutase (SOD) and immunoglobulin A (IgA), suppressing malondialdehyde (MDA) and myeloperoxidase (MPO), decreasing tumour necrosis factor (TNF)-α, interleukin (IL)-1 and IL-6 levels via inhibition of nuclear factor (NF)-κB transcription in IBD rats.

Administration of JPQCHSD suppressed TNBS-induced IBD in rats, presumably by inhibiting the oxidative stress response, neutrophil infiltration and inflammatory cytokines release induced by the overexpression of NF-κB; JPQCHSD also increased serum the content of immunoglobulin A by affecting the immune system in IBD rats, which may be contributors to the beneficial effects of JPQCHSD in the treatment of IBD (Figure 8). These results suggest that JPQCHSD has healing function on IBD and is a new candidate for the treatment of IBD. Further clinical trials are needed to demonstrate its efficacy and tolerance to IBD.

## Data Availability

All data generated in this study to support the conclusions are included in this article.
